# Doctor-patient care relationship in genetic cardiomyopathies: An exploratory study on clinical consultations

**DOI:** 10.1371/journal.pone.0236814

**Published:** 2020-08-05

**Authors:** Chiara Fioretti, Elisa Magni, Fausto Barlocco, Alessia Tomberli, Katia Baldini, Jodie Ingles, Andrea Smorti, Iacopo Olivotto

**Affiliations:** 1 Department of Education, Languages, Intercultures, Literatures and Psychology, University of Florence, Florence, Italy; 2 Cardiomyopathy Unit, Careggi University Hospital, University of Florence. Florence, Italy; 3 Cardio Genomics Program at Centenary Institute, The University of Sydney, Camperdown, NSW, Australia; King's College London, UNITED KINGDOM

## Abstract

**Background:**

The present study aims to explore the setting of consultation and communication between physicians and patients affected by genetic cardiomyopathies, investigating how the two parts of the therapeutic relationship participate and share information.

**Methods and results:**

45 adult patients affected by various cardiomyopathies took part in a prospective case study while attending consultations at a cardiologic outpatient clinic constituting an Italian referral centre for cardiomyopathies. A researcher observed the consultations, which were audio-recorded and transcribed. Transcripts were coded and an analysis of setting, type of communication implemented and participation of doctors and patients in terms of word-count and type of questions/answers was carried out.

Overall word-count was significantly higher for physicians than for patients (*t*(44) = 9,506; *p*<0.001). Doctors were prone to ask closed questions (*t(*44) = -11,90; *p*<0.001) while patients preferred open answers (*t*(44) = 5.58; *p*<0.001), enriched with subjective issues related to their illness experience. Partial correlation highlights a significant positive relation between doctors’ closed question and patients’ open answers (*r* = .838; *p*<0.001).

**Conclusions:**

Findings emphasize patients’ need for adequate time and space to share their subjective illness experience with the physician, within an approach informed by the insights and recommendations of Narrative Medicine. These findings are instrumental to improving the specific clinical setting for individuals with genetic cardiomyopathies.

## Introduction

Narrative Medicine can be defined as the theoretical and operational perspective that aims to include the use of narrative as a tool for collecting and interpreting information on the patient's experience of illness within daily medical practice [1, 2]. In the clinical context, narratives of illness enunciated by patients can be an important tool to help physicians explore symptoms, define a diagnosis and make decisions among different therapeutic options [3, 4]. Scholars following the Narrative Medicine approach have focused strongly on communication in medical practice, observing how doctors' and patients' narratives occupy different positions within clinical consultations, not only due to a different vocabulary but also for the presence of different goals and a diverse interpretation of treatment and care. Such discrepancies are frequently a cause of miscommunication [5].

Patients openly report a need for clear and simple information about their disease and treatment opportunities, but also for openness to dialogue, participation in key decisions and effective emotional support. Notably, patients are more satisfied and ask fewer questions when their doctors show interest and participation in their illness history [6–8]. On the other hand, doctors appreciate those patients who are able to clearly express their symptoms, with whom they feel able to practice competently and effectively [9, 10]. Other studies suggest that doctor-patient communication is more effective when information is shared and decisions are made together [11]. The shared decision- making (SDM) approach uses a patient-centered vision that aims to integrate the biological and clinical dimension with the subjective perspective of the patient. Patients can find relief in dealing with important decisions that affect their health by co-participating in the decision-making process and sharing information about their illness perception; in turn, doctors can feel relief by tailoring treatment decisions with the patient [12, 13].

Genetic cardiomyopathies, defined as “a myocardial disorder in which the heart muscle is structurally and functionally abnormal, in the absence of coronary artery disease, hypertension, valvular disease and congenital heart disease sufficient to cause the observed myocardial abnormality” [14], represent a privileged setting for the implementation of such interventional strategies. Patients with cardiomyopathies are characterized by young age at onset, low mortality and very heterogeneous impact on well-being and quality of life, ranging from functional limitation to atrial fibrillation and heart failure, to risk of premature sudden cardiac death, requiring a long-term relationship with a dedicated healthcare team [15, 16]. Studies on patients’ subjective experience of cardiomyopathies show a wide range of concerns which can be largely associated with the risk of progression over time. For instance, patients express concerns regarding the future of one's abilities, as well as about the financial burden of specialist care and the ability to maintain employment [17]. In addition, the fear and uncertainty associated with the genetic nature of the diseases generate additional concerns for the family members including offspring. Therefore, the hereditary nature of the pathology and the uncertainty of the prognosis lead the person to face important treatment decisions, such as having to undergo defibrillator implantation, cardiac surgery or transplantation [17].

In this perspective, consultations and management decisions are strongly related with patients’ illness narratives and require an open and shared communication between the two parties in the doctor-patient relationship. Scholars report the need to debate several issues related to physical and psychological consequences within consultations [18]. For instance, doctor-patient communication in genetic cardiomyopathies needs to focus on both individual and family implications involved in the living with and managing a chronic disease, and the importance of dedicating part of the consultation to subjective and intrapsychic illness experience has been widely remarked upon [19]. Due to these characteristics, the care of cardiomyopathies requires a multidisciplinary approach, involving not only nursing and medical care, but also genetic and psychological counselling, situating the healthcare team-patient encounter in a complex environment involving several professionals.

This implies the health staff needs to develop tools to facilitate, understand and interpret communication flow. Whether this is the case, and how this can be improved remains largely unexplored.

We performed an observational study aimed at exploring and understanding the complexity of communication between cardiologists and patients affected by cardiomyopathies, as well as the setting of consultation. These, in the light of the theoretical and operational approach of Narrative Medicine, are the means through which objective and subjective information about the experience of disease are integrated and interpreted. To this aim, the following variables were assessed:

- who were the actors involved in the consultation and the characteristics of the setting within which the doctor-patient encounter takes place;
- the type of communication in terms of:
  - communication flow among participants in consultations;
  - roles and dynamics of participation in the medical consultation, with specific reference to the active or passive role of the patient and the physician;
  - type of questions and information shared, with specific reference to open and closed questions within the consultation.

## Methods

The study took place between January and April 2019 at a single centre, the outpatient clinic of a cardiology department with a unit specifically dedicated to cardiomyopathies within one of the largest university hospitals of Italy. The clinic runs regular first and follow up visits to a large number of patients, and provides genetic screening and counselling, which is however managed by the genetics department and which was left out of the present study.

Enrolment took place consecutively one day per week. Patients meeting inclusion criteria were approached by the research staff and invited to participate while they were waiting for the consultation. Only one individual declined. Once informed consent was collected, consultations were audio-recorded in the clinic. Recordings were then faithfully transcribed, anonymized and stored for analysis.

Before patient enrollment, researchers performed a pilot phase dedicated to observing the clinical environment and familiarizing with the hospital staff. A researcher trained in Narrative Medicine approach took part in 10 consultations as an observer. This phase allowed health professionals to familiarize themselves with the presence of a researcher within the consultation setting, in order to reduce possible bias affecting the recordings. Following approval of the research project by the Ethical Committee of Careggi University Hospital (Protocol n. 13916_oss), the data collection phase started and lasted three months. Forty-five patients (28 males, 17 females, mean age 51±18 years) attending the Cardiomyopathy Unit of our Institution participated in the present study. Inclusion criteria were being aged 18 years or more, providing written informed consent and undergoing a consultation for a genetic cardiomyopathy. Most (83%) had a diagnosis of hypertrophic cardiomyopathy (HCM), while a minority (17%) had dilated cardiomyopathy (DCM). Thirty-one of the 45 patients were accompanied by a caregiver (partner, parents or siblings).

Because of the characteristics of the clinic and its location within a university hospital, visits were carried out by a variety of physicians, including the professor responsible for the unit, various cardiologists and some cardiologists in training, some of whom performed the visit as main doctor with the supervision of a senior, who might be moving between different consultation rooms in a given period, and there were always a few cardiologists in training assisting and helping the visit.

### Data coding

The following categories were used to identify the actors and the relevant characteristics of the clinical interview setting (consultation):

1. Actors: the total number of people present in the room at any time during the visit;
2. Movements: the number of people entering and leaving the room at any time during the visit;
3. Dialogues: the total number of "dialogues" during the consultation;
4. Front Stage (FS): the number of dialogues related to the direct interaction between the patient and the other actors present;
5. Back Stage (BS): the number of dialogues in which the actors interact with each other not directly involving the patient. Reference is made, for example, to all those situations in which doctors talk to each other, nurses, other staff members or accompanying persons.

To understand the flow of communication, further variables were identified:

6 Front Stage (FS) was divided into two sub-categories: FS Doctor-Patient and FS Others-Patient. FS D-P refers to the number of dialogues related to the direct interaction of physicians (consultant, staff cardiologists, trainees) with the patient; FS O-P refers to the number of dialogues related to the direct interaction of the patient with other staff members (nurses) or accompanying persons (spouse, children, siblings, etc.). Moreover, the ratio between Front Stage and Back Stage was calculated.

In order to understand the role and dynamics of participation in the medical consultation, a word-count operation was carried out. The language production of doctors and patients was counted and three variables were considered:

7 General Word Count: numerical count of the words spoken by all the actors preset;
8 Doctor's Word Count: words spoken by physicians;
9 Patient Word Count: words spoken by the patient.

Finally, in order to understand the type of queries and information shared within the consultation, both by the physician and the patient, questions and answers were classified as follows:

10 Open questions: the question does not present a choice between two or more fixed options, but rather presupposes an answer that adds free information chosen by the recipient. An example of an open question is "how do you feel?", or "what symptoms have you felt during the last period?”
11 Closed question: the question presupposes an answer that is limited to a choice between the options presented in its statement. Examples of closed questions are: "do you feel well?" (“yes” or “no”), or "have you felt dizzy lately?";
12 Open answer: an answer that provides additional information and argumentation (e.g.: "I have been feeling generally well, although especially in the evening I am sometimes short of breath after meals");
13 Closed answer: an answer limited to one of the options offered, which does not provide additional explanations to what was asked or what is already known. (e.g.: "no, I did not feel that symptom").

With reference to questions asked by the physician, the following distinction was also made between relaunch and new questions:

14 Relaunch questions: questions that relaunch what the recipient says. They serve the scope of deepening or rephrasing what the patient has said. An example of a re-launch question could be: "if you did not feel that symptom, then maybe you felt this one instead?”;
15 New questions: those that introduce a new topic, moving the conversation from one topic to another. The dialogue is of the "knock and answer" type.

Two researchers, CF and EM, individually carried out a preliminary analysis on a sample of 15 transcriptions, which made it possible to define a high degree of inter-judicial agreement of data analysis (K of Choen = 0.94).

Data of the full sample were coded and analyzed by version 25 SPSS data analysis software by means of descriptive statistics, Student t tests and bivariate and partial correlations.

## Results

### The setting

As a first analysis, descriptive statistics of coded variables were verified. Table 1 shows means and standard deviations of variables related to the setting and flow of communication during consultations. At each visit, the number of participants (actors) in the clinic was 8.3±2.5, totaling an average of 12.4±5.9 movements (either entering or leaving the room). Excluding patients, family caregivers and the researcher, there was an average number of 5.3 professionals in the room, including staff and trainees. As pointed out in the familiarization observations, the consultation is a complex context in which several professionals “appear”, sometimes playing a specific role in patient care (e.g. the nurse performing the ECG), sometimes as a rapid transition not related to the visit (a professional looking for a piece of equipment or a colleague).

**Table 1:** Distribution of variables describing the setting of consultation and exchange of information (mean±standard deviation of the number of events per each visit).

	Mean	Standard Deviation
Number of actors	8.3	2.5
Number of movements	12.4	5.9
Number of total dialogues	455.8	181.6
Frontstage dialogues	328.6	158.9
Backstage dialogues*	128.4	92.9
Doctor-Patient Frontstage	299.8	147.9
Others-Patient Frontstage	27.4	22.00
General Word-Count of the consultation	3994.2	1646.6
Doctor Word-Count	2629.4	1167.9
Patient Word-Count	1053.3	662.5

*Not normally distributed. Median (89.50) and Interquartile range (83.51) are reported.

### Communication flow

With respect to how professionals and patient manage the flow of communication, we found that 72.1% of the total number of dialogues were frontstage, i.e. directly connecting the healthcare worker with the patient, while 28% were backstage dialogues, in which the patient was not directly involved.

The word count carried out on consultation transcripts showed that 65.8% of words were spoken by physicians, compared to 26.3% by patients and 7.9% by other participants (family members, other professionals). These results underline how the doctor and the patient represent the absolute protagonists of the consultation, as appropriate, although with a clearly preponderant role of the former. From a verbal point of view, other participants seemed to play a secondary role within the consultation setting.

Furthermore, the number of Front Stage movements during the visit was significantly higher than the number of Back Stage (BS) dialogues (t(44) = 7,262; p<0.001), although BS still accounted for one out four moves.

The consultation of patient n. 2.06, a 35-year old male, offers us a clear example of BS dialogues and their impact on patient’s experience:

[Doctor 1 is performing echocardiography and is commenting results with a student]

Doctor 1: did you measure it…?

*Student*: *Yes I did…both here and there…here it’s 35, there 34…*

[sounds from the machine]

*Doctor 1*: *maybe the acoustic window can be improved… (to the patient:) Sir, can please you inhale some air? Ok…like this…perfect! Now breathe normally! (To the student:) Ok, now measure it, this is the right point!*

[nurse 1 touches the screen and say something to doctor 1]

*Doctor 1*: *here, at this point.. it’s somewhat dilated. . . . we have to check if the heart presents an abnormal flow here, within the left ventricle*

*Student*: *mmm mmm…*

*Patient*: *is everything ok?!*

(Extract n. 1)

Comparing the two components of FS interactions, physician-patient contacts were largely predominant over others-patient interactions (t(44) = 12,985; p<0.001). Thus, despite the high number of actors within the clinical interview, doctors and patients undoubtedly remain the main protagonists of the consultation, with limited collateral activity. In this context, the word count analysis limited to FS interactions reiterates a preponderance of words spoken by the physician versus those by the patient (t(44) = 9,506; p<0.001): in the relationship between the two protagonists of the consultation, the doctor seems to have a greater weight and a wider narrative space.

### Questions and answers

**Fig 1:**
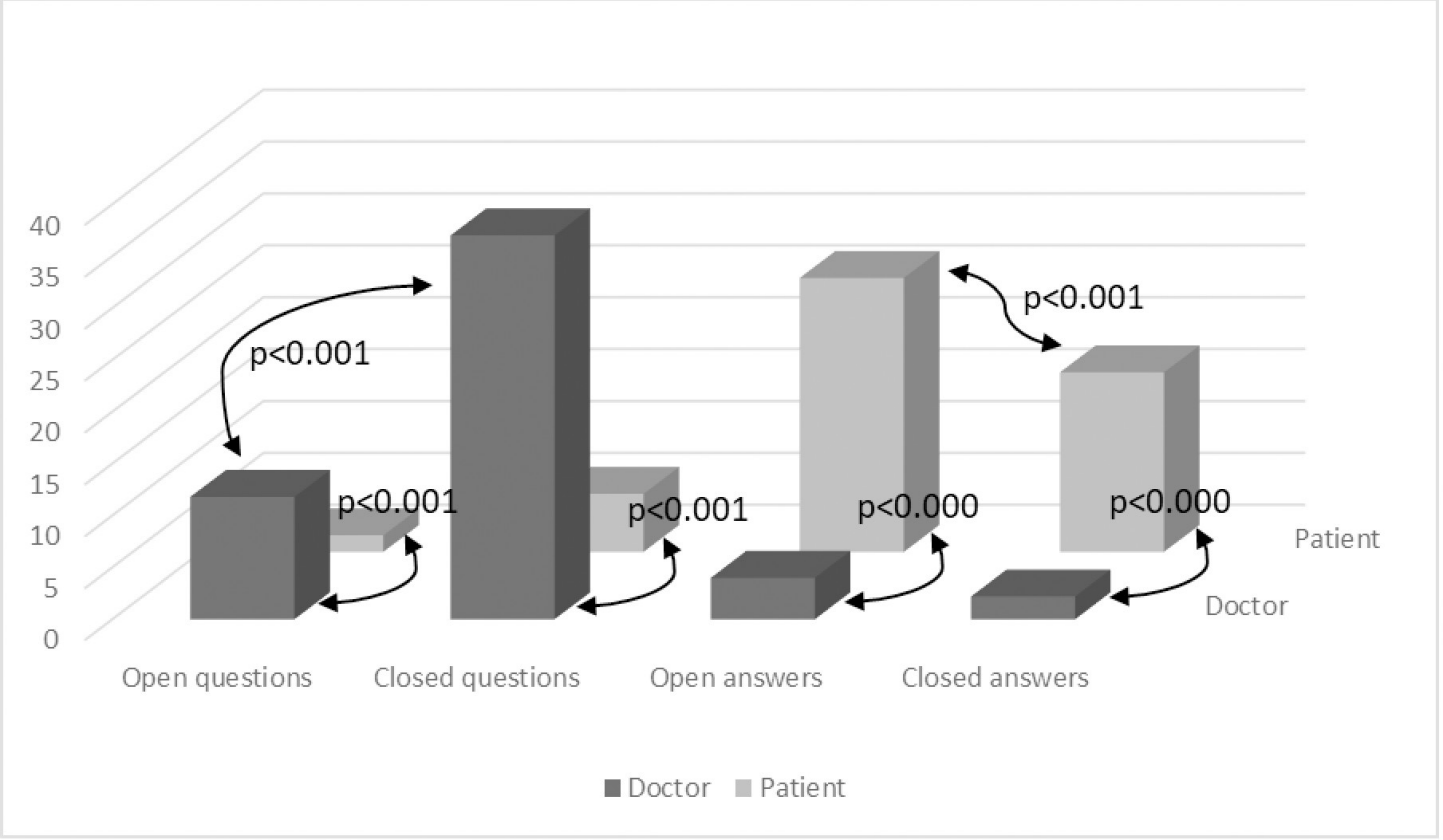
Means and p value of Student t test comparing doctors and patients’ open and closed questions and answers and p value.

During the visit, physicians were far more prone than patients to ask questions (an average of 48.7 questions per consultation, compared to 7.26 questions for patients), both of the open and closed type (t(44) = 9,02; p<0.001 and t(44) = 11,25; p<0.001, respectively). Fig 1 shows results of word count and questions/answers comparison between doctors and patients.

Of note, doctors were more likely to ask closed rather than open question (t(44) = -11,90; p<0.001). Furthermore, doctors more often used relaunch rather than new questions (*t*(44) = 2.189; *p* = .03): physicians most often started from patients’ information and previous answers to formulate their questions rather than proposing new issues bypassing their interlocutors’ narratives. The following extract of consultation of patient 1.09 (a 51-year old female) provides an example of consultation focused on relaunch questions:

*Doctor 2*: *Okay… Good morning Mrs X.*

*Patient*: *Good morning*

*Spec2*: *I’m Dr. Y.*

*Patient*: *Good morning Doctor*

*Spec2*: *How are you?*

*Patient*: *yes… now I am quite well in the sense that I am already taking bisoprolol and the cardiologist has increased it to 3.75 because I kept having this feeling of heaviness on my chest, more than anything else… a lot of extrasystole*

*Spec2*: *how do you feel the extrasystoles?*

*Patient*: *Well… the extrasystoles have already been there for some years and the doctor at my job place found it, during a routine visit, then I did all the exams, the Holter and so on, and they found I had 13,000 per day with couplets and triplets and I started to take medicines. The bisoprolol, a little bit but I took 1.25 mg per day… it seems to me per day… then lately I was… I feel just my heart that… like the heart in my throat… bum bum bum… strong… and then sometimes the feeling stopped, sometimes it continued…In short, I went back to the cardiologist and the extrasystoles had increased in fact and so we increased the quantity of bisoprolol, then I did the MRI that I sent you some time ago and now I am quite well… but every now and then I get a heaviness also in breathing, difficulty in breathing deeply… maybe when I go shopping…*

*Doctor 2*: *what about during physical exercises?*

*Patient*: *no no no… even in normal conditions… maybe I'm at work so I feel like this… I don't know if it’s just a little anxiety or if it's due to this… this fact of the extrasystoles… I don't know, because I feel like this… a little tachycardia… something that beats inside […]".*

(Extract n.2)

In order to investigate the potential impact of Back Stage interaction on patients’ propensity to ask questions, we ran a bivariate correlation between the FS-BS ratio and the number of patients’ open and closed questions. Results indeed showed that in the presence of greater proportions of BS in respect to FS interactions, patients were less likely to interrogate their physicians (r = -0.479, p<0.001).

As a consequence of doctors asking most of the questions, patients are the interlocutors spending more time and words in answers, both in the open (t(44) = 9.67; p = .000) and closed form (*t*(44) = 11.608; *p* = .000). Intriguingly, while doctors more often asked closed questions, patients clearly preferred open answers (t(44) = 5.58; p<0.001). In order to further investigate the relationship between doctors’ questions and patients’ answers within the therapeutic relationship, a bivariate correlation has been carried out (see Table 2). The analysis showed a positive correlation between the doctor's open questions and both the patient's open and closed answers.

**Table 2:** Bivariate correlations among doctor’s questions and patient’s answers.

	Patient’s open answers	Patient’s closed answers	Doctor’s open questions	Doctor’s closed questions
Patient’s open answers	-----------	.767*	.848**	.856**
Patient’s closed answers	.767*	-----------	.710**	.765**
Doctor’s open questions	.848**	.710**	-----------	.651*
Doctor’s closed questions	.856**	.765**	.651*	-----------

*p < .05

**p < .01

Furthermore, a partial correlation was run to test the relationship between doctor's closed questions and patient's open answers, controlling for the doctor's open questions and the patient's closed answers, in order to control for the interference of the other types of questions and answers by the two protagonists. Indeed, when doctors asked their patients closed questions, patients had a strong tendency to answer with open narratives, sharing details, symptoms and other considerations about their illness. This correlation remained true after controlling for the intervening variables (*r* = .838; p<0.001). Patient n. 3.07, a 75-year old female, provides an example of open-ended answers to closed questions:

*"Doctor 1*: *Is your blood pressure low at home? Do you measure it?*

*Patient*: *no I hardly ever measure it… I'm a bit… dizzy…like this…*

*Doctor 1*: *Are you dizzy?*

*Patient*: *no or… rarely*

*Doctor 1*: *For example like when you get out of bed?*

*Patient*. *Yes… in the morning… in the morning sometimes, even down the stairs… because we have rooms upstairs, so I need to go upstairs often and it’s very hard for me… sometime ago I fell… I fell! Nothing important, but I fell…"*

(Extract n.3)

## Discussion

We investigated the setting and the type of communication implemented in consultations between patients suffering from cardiomyopathies and their physicians in a specialized multidisciplinary cardiology clinic.

From the patients’ perspective, such interpersonal complexity and the number of participants and movements involved could be a source of confusion and incomprehension with the health provider. This critical point is suggested by the relevant percentage of backstage dialogues during consultations, involving one out four interactions during the visit originating from other participants in the room and not directed to the patient. Indeed, we observed that higher proportions of backstage interactions correlated inversely with the likelihood of patients asking questions, suggesting a negative impact on their active role during the visit. The effect of backstage activity on doctor-patient alliance during outpatient visits requires further investigation.

**Fig 2:**
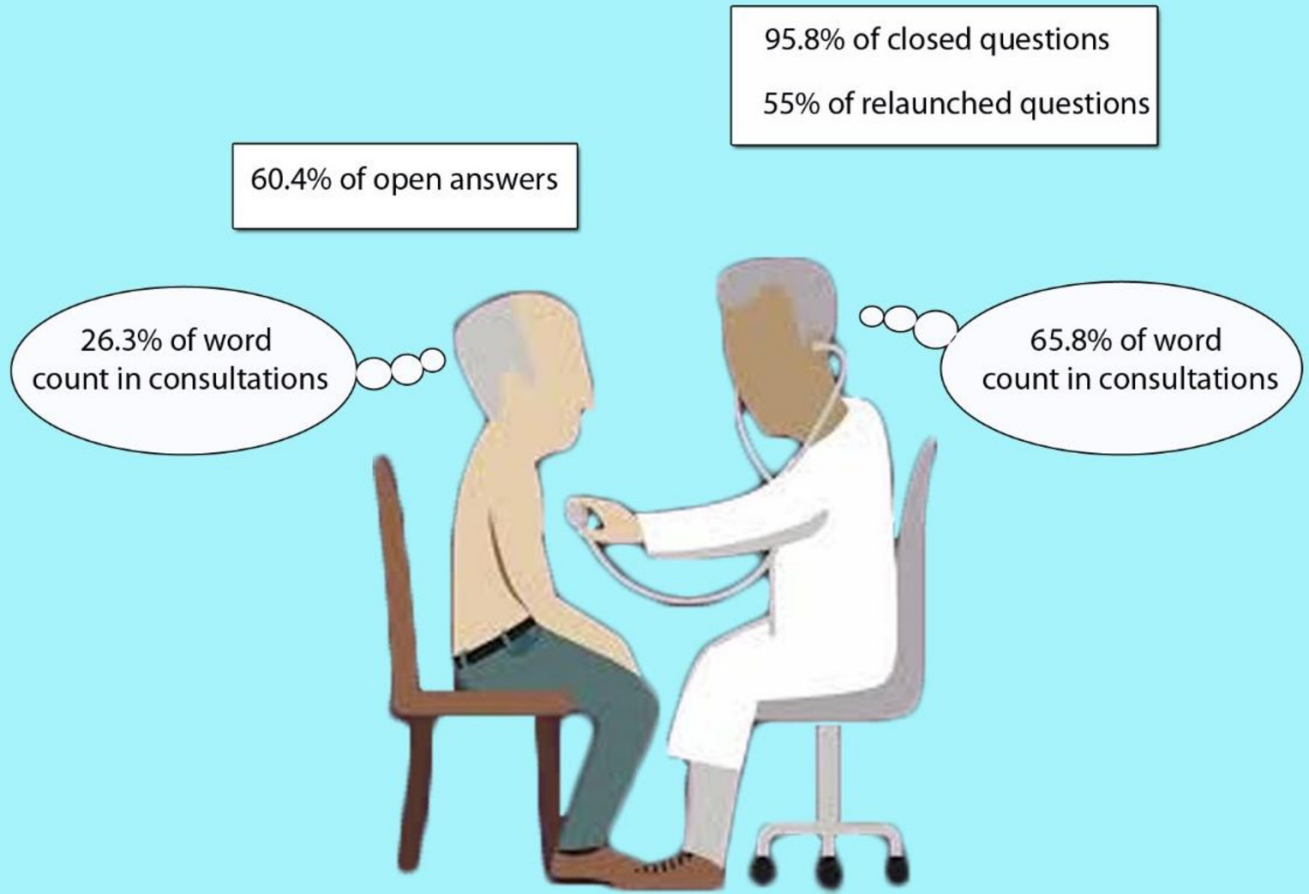
Summary of main results on communication flow in observed consultations.

Despite this potential confounder, frontstage relationships with patients were largely preponderant, particularly–as expected–those involving a direct dialogue with the attending physician. This finding suggests that, despite the technical and procedural complexities involved in the consultation, the privileged patient-physician relationship is appropriately preserved. However, within this dyadic relationship most of the communication space is occupied by the doctor, as highlighted by word-count analysis, suggesting that patients may not always have the chance to narrate their illness experience extensively. This limit may also affect the empowerment process in clinical decision-making that is so relevant to chronic illnesses such as cardiomyopathies. The large gap between doctor and patient word-counts suggests the need to reflect on patient involvement during visits, allowing more time and opportunities for balanced bilateral communication. To fill this gap in communication, professionals could reserve a space in consultation for patients’ narrative of illness allowing them to bring in the discussion subjective issues related to the individual experience of suffering for genetic diseases. Studies on doctor-patient communication in fact underline that collecting patient’s narrative may improve the quality of care of chronic disease [20]. Since time is currently one of the major problems of professionals working in hospital environments, narrative could be connected by means of face to face or telephone interviews before consultations by nurses or social workers such as psychologists or counselors. A growing number of reports highlights that when patients are actively involved in their care, adherence to treatments and therapeutic alliance are improved, with objective benefits [21]. In the specific case of cardiomyopathies, most patients are involved therapeutic programs often focused on a single annual consultation. Between visits, doctor-patient relationships are based on contacts by telephone or e-mail, with strong emphasis on patients to actively manage daily health issues and monitor their health conditions. When patients meet their physicians for follow-up consultations, especially when these are relatively infrequent, they exhibit a strong need to share illness experience finding the appropriate space to narrate and ask questions relevant to their health condition [1, 2]. Fig 2 summarizes main results of the present study.

The present study provided intriguing insight into the preferred dialectic modalities of the two protagonists during visits.

Physicians clearly preferred closed question, likely aimed at obtaining rapidly the most relevant clinical information regarding patients’ health, including symptoms, recent events and current medical therapy, rather than a narrative of the patient's illness that could provide more subjective information. This objective is in line with the strictly medical aspects of cardiomyopathies, focusing on the information the physician believes useful to reach a correct diagnosis and to define a tailored treatment program. Nevertheless, patients were consistently providing open answers to the closed questions by their physicians. This was shown by the higher Pearson score of correlation of open compared to closed answers with closed questions. In the face of punctual questions from the health provider, patients irresistibly tend to narrate more subjective issues, express doubts and provide additional details. In line with the Narrative Medicine approach [4], these results suggest that patients need an appropriate space to narrate their experience and bring their subjective point of view on disease management. Taking this narrative space, patients not only provide examples related to everyday life by reporting information that may be useful to the doctor, but also take the opportunity to narrate their interpretations of the possible causes of the experienced symptoms and describe the evolution of their perception of illness over time [1].This narrative effort, as discussed at length in a convincing way in much medical sociological and anthropological literature [22–24], by situating the illness in the patients’ biographies, helps them make sense of their experience of chronic illness or its extreme manifestations by helping answering questions such as ‘why this?’ and ‘why to me?’.

Patients’ attitude to provide open answers may also be empowered by the use of re-launch questions. Our study showed that physicians are prone to formulate questions starting from information provided by their patients, facilitating patients’ perception of having something important to share about their illness story. This type of question gives the idea of a fluid dialogue, in which the doctor uses instruments that are brought by the patient to deepen the visit and enrich the anamnesis and decisional process. The patient, consequently, perceives doctors’ interest for their illness narrative. Relaunch questions give patients the opportunity to bring in the discussion more details about their subjective symptoms, by perceiving their physician’s interest on their point of view, as shown by extract number 3. From the other side, the doctor follows the patient’s narrative and stimulates it with questions related to the subjective issues offered. Consequently, as emphasized by theorists of Narrative Medicine, patients have the opportunity to be protagonist of their own story [2, 25].

### Limitation and future studies

The present paper discusses the results of an observational study and therefore the limits associated with this design should be considered. If, on the one hand, the study provides a novel snapshot of the events of interest in a given instant, useful to estimate behaviors, interpret meanings and generate hypotheses, it does not allow to generate causal conclusions. In addition, data are based on a relatively small patient sample, which may not be fully representative of the entire population of patients attending a Cardiomyopathy Unit. Other aspects to keep in mind are related to possible bias due to the presence of an external researcher during visits, even though a period of familiarization was carried out in order to reduce potential distortion in patients’ and physicians’ attitude to manage the recorded interview. Furthermore, due to time and resources limitation, patient satisfaction has not been investigated in the project described here, but it is the subject of an intervention study that builds on our findings. Future studies should also consider different roles of cardiologists in consultations, considering potential differences between senior and trainee doctors. Furthermore, while our work was limited to doctor-patient communication, since patients with cardiomyopathies are involved in several therapeutic relationships with different professionals, including genetic counselors, studies in these specific settings of consultation should be performed.

Moreover, due to its limitations in establishing causality and more generally its design, the study has not attempted to measure or specify the possible effects of the settings and characteristics of communication observed on therapeutic interventions and goals, such as, for instance, different level of compliance or adherence to treatments, number of subsequent hospitalizations or forms of stress.

In conclusion, the consultation for cardiomyopathy patients at the outpatient clinic object of study took place in a complex environment, centered around the doctor-patient relationship, but with relevant multidisciplinary background interaction that may affect patients’ propensity to actively participate in the dialogue. As doctors tend to speak more and prefer closed questions, patients tend to answer through open answers, suggesting their need to provide their personal perspective on illness. Cardiomyopathies have diverse and profound implications on patients’ health and quality of life, creating complex needs and expectations in patients when they meet their health providers. In the light of a Narrative Medicine approach, our findings emphasize patients’ need for adequate time and space to share their subjective illness experience with the physician [26]. These findings are instrumental to improving the optimal clinical setting for individuals with genetic cardiomyopathies.
